# Safety and Efficacy of Tenofovir Alafenamide Fumarate in Early-Middle Pregnancy for Mothers With Chronic Hepatitis B

**DOI:** 10.3389/fmed.2021.796901

**Published:** 2022-01-17

**Authors:** Ruochan Chen, Ju Zou, Liyuan Long, Haiyue Huang, Min Zhang, Xuegong Fan, Yan Huang

**Affiliations:** ^1^Department of Infectious Diseases, Xiangya Hospital, Central South University, Changsha, China; ^2^Hunan Key Laboratory of Viral Hepatitis, Changsha, China; ^3^Yali High School International Department of Changsha, Changsha, China; ^4^National Clinical Research Center for Geriatric Disorders, Xiangya Hospital, Central South University, Changsha, China

**Keywords:** chronic hepatitis B, tenofovir alafenamide fumarate, early, pregnancy, mother-to-child transmission

## Abstract

**Background:**

Tenofovir alafenamide fumarate has been used in late pregnancy; however, no data exist regarding its safety and effectiveness in early and middle pregnancy for mothers with hepatitis B virus infection.

**Aims:**

To design a prospective study to investigate the efficacy and safety of TAF in pregnant women with chronic HBV infection during early-middle pregnancy.

**Methods:**

Pregnant women with active chronic hepatitis B who received tenofovir alafenamide fumarate during early and middle pregnancy were enrolled and followed up until 6 months postpartum. Infants received immunoprophylaxis. The primary endpoint was the safety of mothers and infants. The secondary endpoints were maternal hepatitis B virus DNA reduction at delivery and mother-to-child transmission rate.

**Results:**

Among 98 mothers enrolled, 31 initiated tenofovir alafenamide fumarate in early pregnancy, and 57 in middle pregnancy. The mean (± standard deviation) age was 29.00 (±3.81) years. At delivery, 100% (98/98) of the mothers achieved hepatitis B virus DNA levels <200,000 IU/L. Ninety-eight infants were born, and none had congenital defects or malformations. All infants received hepatitis B virus immunoprophylaxis. The mother-to-child transmission rate was 0%. Growth parameters including body weight, height, and head circumference were comparable to the national standards for physical development. No severe adverse effects were reported in either mothers or infants. No severe liver function damage occurred in any of the mothers.

**Conclusions:**

Initiating tenofovir alafenamide fumarate in early and middle pregnancy appears safe for both mothers and infants, and it is effective for controlling maternal disease as well as interrupting mother-to-child transmission.

## Introduction

There were an estimated 257 million chronic hepatitis B virus (HBV) carriers worldwide in 2015, and 887,000 people died due to HBV-related complications, which mostly included end-stage liver diseases such as cirrhosis and hepatocellular carcinoma (1). Thus, in May 2016, the World Health Organisation developed the “Global health sector strategy on viral hepatitis 2016–2021,” which aims to eliminate viral hepatitis by 2030. Currently, an estimated 4.5 million women with chronic HBV infection give birth annually, with the largest number living in Africa and Western Pacific regions (2). Mother-to-child transmission (MTCT) is the most common route of HBV transmission, and is thus the key target for controlling HBV infection. Standardised management of mothers and infants is an effective measure to control MTCT of HBV (3, 4). However, despite years of continuous and unremitting efforts, we still face challenges.

Prevention of perinatal transmission relies on reducing high HBV DNA levels among mothers and administering timely immune prophylaxis. In addition, mothers require appropriate antiviral treatment to prevent liver dysfunction or even liver failure due to HBV active infection. Currently, the major association guidelines recommend tenofovir disoproxil fumarate (TDF) as the first-line therapy for mothers due to a high gene barrier and low drug resistance rate (5–8). However, several studies have reported negative effects on bone mineral density and neutropenia in early age infants with foetal exposure to TDF (9, 10). Tenofovir alafenamide fumarate (TAF) is a recently developed prodrug of tenofovir similar to TDF that has an improved renal and bone safety profile compared to that of TDF, while maintaining similar virological efficacy and safety (11, 12). TAF has been approved and recommended as the first-line therapy for HBV by medical associations in Japan, Europe, America, and China for the treatment of chronic hepatitis B (CHB) patients (6, 13).

There are extremely limited data regarding the role of TAF in preventing MTCT in pregnancy (14, 15). Moreover, mothers with CHB present a unique dilemma for clinicians, since the management approach for this population must consider not only the reduction of MTCT but also control of maternal disease progression, especially when dealing with mothers with active CHB in early pregnancy. However, there are no clinical data on the use of TAF in early-middle pregnancy for controlling maternal disease and preventing MTCT.

Thus, we designed a prospective study to investigate the efficacy and safety of TAF in pregnant women with chronic HBV infection during early-middle pregnancy. Our study provides preliminary data to support that TAF treatment is a safe and effective option for pregnant women in early middle pregnancy and contributes to the knowledge of antiviral therapy in this special subgroup of the population.

## Methods

### Patient Selection

This was a prospective study. Patients were recruited from Xiangya Hospital, Central South University of China between 1 January 2019 and 30 January 2021. The study was approved by the Institutional Ethics Review Committee of Xiangya Hospital. The study was conducted in accordance with the guidelines of the Declaration of Helsinki and the principles of Good Clinical Practise. TAF has been approved for the treatment of chronic hepatitis B in China since December 2018, and it has the indication for pregnant mothers stated clearly in the instructions. Informed consent was obtained from all patients before recruitment into the study, and all patient data were anonymised.

Pregnant mothers with chronic HBV infection in early and middle pregnancy were included if they fulfilled the following eligibility criteria: (1) they were aged above 20 years; (2) they had active CHB, classified as alanine aminotransferase (ALT)≥2 upper limit of normal value (ULN) more than two times during 1 month and HBV DNA >100,000 IU/ml (16); (3) they were in early pregnancy, defined as <14 weeks of gestation, or middle pregnancy, defined as between 14 and 28 weeks of gestation (17). The indication for antiviral therapy was the enrolment of mothers with active CHB. The exclusion criteria were as follows: (1) co-infection with HAV, HCV, HDV, HEV, HIV, syphilis, or *Toxoplasma gondii*; (2) use of other HBV antiviral drugs as monotherapy or in combination with TAF during pregnancy; (3) participation in other clinical trials and use of investigational regimens; (4) evidence of hepatocellular carcinoma, cirrhosis, and other liver diseases such as autoimmune liver disease, Wilson's disease, alcoholic liver disease, or drug-induced hepatitis; (5) receiving chemotherapy or immunosuppressive therapy such as immune modulators, cytotoxic drugs, or steroids.

### Study Design and Data Collection

**Figure 1:**
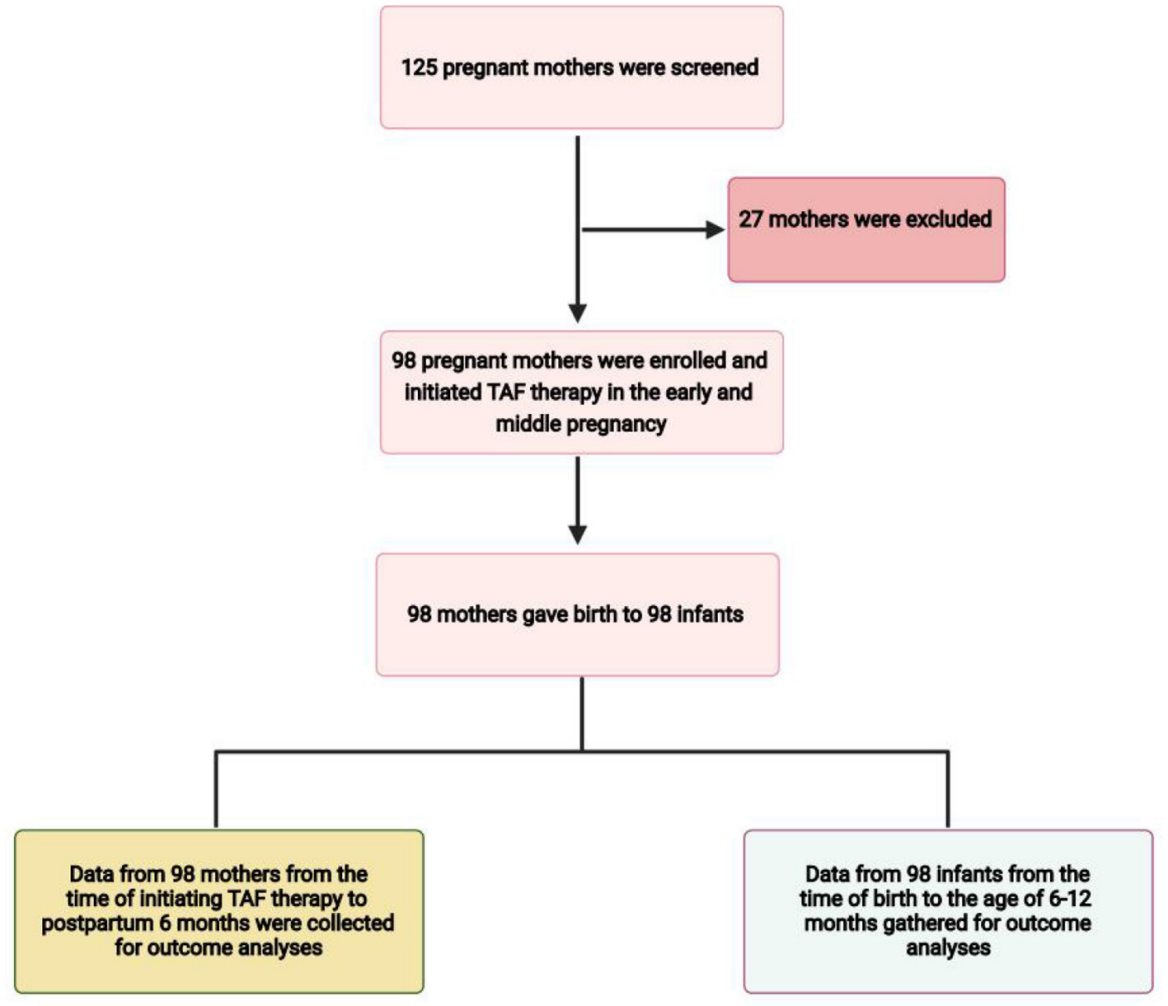
Flow diagram of the study.

Pregnant women with CHB who met the inclusion criteria were selected, and medical data were extracted from their medical records (Figure 1). All mothers continued TAF therapy after delivery. Every mother was closely followed up in the outpatient department of our hospital, and prenatal drug safety consultations were routinely provided by specialists, and alternative options were discussed. Blood tests were performed at the laboratories of Xiangya Hospital. We collected patient data from medical records, and all data were de-identified before analysis. Maternal data during pregnancy and 6 months postpartum included demographic data, gestational age at initiation of TAF therapy, duration and adverse events of TAF therapy, gestational age at delivery, serum ALT levels, HBV DNA, and HBV serologic markers such as hepatitis B surface antigen (HBsAg) and hepatitis B e antigen (HBeAg) positivity. The infants' data included birth weight, birth length, Apgar score at birth, physical growth measurements from birth to the age of 6 months, details of immunoprophylaxis administration for HBV and HBV virological testing at birth, and completion of the three hepatitis B vaccination doses (at age 7–12 months).

### Outcome Measurements

Outcome measurements included a safety assessment and effectiveness evaluation. The primary outcome was the safety of TAF therapy for mothers and infants. Maternal safety assessment mainly included perinatal adverse events, complications, renal function impairment, and ALT flares at delivery and 6 months postpartum. Caesarean section rates were also included in the safety analysis (2, 18). ALT flare was defined as a level >5 or 10 times the upper limit of normal (ULN) (19). Infant safety was mainly assessed by infants' congenital defects and physical growth, including weight, height, and head circumference at birth and 6 months according to China's standards (20). The secondary outcomes concerning effectiveness were vertical transmission rates and virological parameters of mothers at 6 months postpartum. Infants with positive HBsAg and detectable HBV DNA were counted as HBV infected cases. The vertical transmission rate was the percentage of infants with HBV infection among all live births in the current study.

### Statistical Analysis

According to the published data for sample size calculation by Ding et al. (15) and Pan et al. (19), the patient sample size needed in this study was calculated to be 70 with a significance level of 0.05 (one-tailed). Thus, 98 patients were enrolled in our study. Continuous variables are presented as means with standard deviations or medians with ranges. Categorical variables are presented as proportions or frequencies. For the quantitative variables, Student's *t*-test was used to compare group differences. For categorical variables, the chi-square test or Fisher's exact test were used for group comparisons. All statistical analyses were performed using SPSS version 25.0 (IBM, Armonk, NY, USA). All tests were two-tailed, and statistical significance was set at *P* < 0.05.

## Results

### Baseline Characteristics of Mothers and Infants at Birth

Among the 125 mothers screened, 98 mothers who met the inclusion criteria were enrolled in the study (Figure 1). All mothers were treated with oral TAF (Vemlidy, Gilead Science, Inc., Foster City, CA, USA) at a dose of 25 mg daily (patients were instructed to take it every 24 h at any time of day each day). The baseline maternal and infant values are summarised in Table 1. The mean (±SD) maternal age was 29.00 (±3.81) years. There were 47 mothers who initiated TAF treatment in early pregnancy and 51 in middle pregnancy. Drug compliance to TAF was assessed by the attending physician at each visit and the medication possession ratio (MPR) was calculated as the total number of days of medication supply divided by the time interval (21). All mothers were compliant with TAF treatment during pregnancy. All mothers continued TAF treatment after the endpoint of observation. All pregnancies were singleton; therefore, the total number of live births from 98 mothers was 98. The maternal and infant baseline values are presented in Table 1.

**Table 1:** Maternal and infant baseline values.

**Variables (mean ±SD, or specified)**	**TAF treated (*N* = 98)**
**Maternal data**	
Early pregnancy	*n* = 47
Middle pregnancy	*n* = 51
Age (y)	29.00 ± 3.81
HBV DNA-Log10 (IU/mL)	7.07 ± 0.91
ALT, U/L (normal ≤ 40)	197.41 ± 114.71
HBeAg positivity, *n* (%)	98/98 (100%)
Duration of exposure, weeks	27.99 ± 3.27
**Infants' data (mean** **±SD, or specified)**	
Gestational age (wks.)	38.46 ± 2.17
Male gender, *n* (%)	41/98 (41.83%)
Pre-term infants, *n* (%)	2/98 (2.04%)
Delivery with caesarean section, *n* (%)	16/98 (16.32%)
Infant height (cm)	50.19 ± 1.68
Infant weight (kg)	3.35 ± 0.29
APGAR score at 5 min	9.85 ± 0.10
APGAR score at 10 min	9.85 ± 0.10
Congenital defects or malformations at birth	0/98

*SD, Standard deviation; HBV, Hepatitis B virus; HBeAg, Hepatitis B e antigen*.

### Safety Evaluation of Infant and Mothers

The mean gestational age of the 98 infants was 38.46 ± 2.17 wks, and two (2.04%) were pre-term infants. Sixteen (16.32%) infants were born via caesarean section. There were no reported congenital malformations or defects at birth in all 98 infants, and no infant had an Apgar score of <8 at 5 min of birth (Table 1). Additionally, no abnormal signs or symptoms were reported among the infants in our follow-up inquiry. The physical parameters stratified by sex and gender of infants at birth and at 6 months were within the normal range, compared with the national children's reference values. The specific data comparisons between the physical growth parameters of infants with TAF exposure and the national standards for infant growth are presented in Table 2.

**Table 2:** Growth parameters of infants with TAF exposure at birth and 6 months.

**Growth parameters, mean (±SD)**	**National children's reference values at birth (*N* = 3,811)**	**Infants at birth (*N* = 98)**	***P*-values**	**National children's reference values at 6 months (*N* = 3,811)**	**Infants at 6 months**	***P*-values**
Boys' weight	3.38 ± 0.40	3.41 ± 0.13	0.459	8.26 ± 0.94	8.32 ±0.78	0.531
Girls' weight	3.26 ± 0.40	3.28 ± 0.27	0.623	7.60 ± 0.85	7.69 ± 0.91	0.302
Boys' height	50.4 ± 1.6	50.25 ± 1.78	0.299	67.7 ± 2.3	67.58 ± 2.58	0.611
Girls' height	49.8 ± 1.6	49.60 ± 1.05	0.219	66.1 ± 2.3	66.19 ± 1.78	0.701
Boys' head circumference	34.0 ± 1.4	34.27 ± 1.08	0.058	42.9 ± 1.3	42.9 ± 1.3	1.0
Girls' head circumference	33.7 ± 1.3	33.61 ± 1.27	0.498	41.8 ± 1.3	41.91 ± 1.26	0.408

*SD, Standard deviation*.

All 98 mothers tolerated TAF therapy well, and there was no withdrawal of TAF due to adverse events. Adverse events and complications were based on a medical record review and are recorded in Table 3. The reported adverse events included headache, nausea, vomiting, stomach ache, constipation, diarrhoea, and skin rash. The reported perinatal complications were hyperemesis gravidarum (3), gestational hypertension (2), gestational diabetes mellitus premature (1), oligohydramnios (2), rupture of membranes (2), and pre-term labour (2). None of the 98 mothers had ALT flares after TAF treatment (*n* = 0/98). No significant change in serum creatinine level was observed between baseline and 24 weeks postpartum (*p* = 0.687). However, there was a significant decline in the mean estimated glomerular filtration rate (eGFR) from baseline to 6 months postpartum (*p* = 0.001; Table 4). No significant changes were observed in the serum phosphorous levels between the baseline (3.41 ± 0.72 mg/dL) and 6 months postpartum (3.35 ± 0.50 mg/dL, *P* = 0.499).

**Table 3:** Maternal adverse events and complications reported in the study.

**Adverse events or complications, *n***	**TAF treated (*N* = 98)**
**Maternal adverse events**
Headache	2/98
Nausea	3/98
Vomiting	1/98
Stomach ache	1/98
Constipation	3/98
Diarrhoea	1/98
Skin rash	1/98
ALT flares	0/98
**Maternal complications**
Hyperemesis gravidarum	3/98
Gestational hypertension	2/98
Gestational diabetes mellitus	1/98
Oligohydramnios	2/98
Premature rupture of membranes	2/98
Pre-term labour	2/98

*TAF, Tenofovir alafenamide fumarate*.

**Table 4:** Maternal renal function and serum phosphate changes in the study.

**Variables, mean (±SD) or specified**	**Baseline**	**Follow-up assessments-6 months post-partum**	***P*-values**
Creatinine (umol/L)	54.88 ± 11.23	55.48 ± 9.54	0.687
eGFR (mL/min/1.73 m^2^)	125.76 ± 13.04	117.74 ± 19.67	0.001
Serum phosphate (mg/dL)	3.41 ± 0.72	3.35 ± 0.50	0.499

*SD, Standard deviation; eGFR, estimated glomerular filtration rat*.

### Effectiveness in Mothers and Infants

The effects of maternal treatment with TAF are presented in Table 5. For all 98 mothers, the decrease in HBV DNA from baseline to delivery was significant (*p* < 0.001). There was also an obvious continual decrease in HBV-DNA from delivery to 6 months postpartum (*p* < 0.001). No mother achieved HBsAg or HBeAg clearance at delivery or at 6 months postpartum. The mean (±SD) ALT was 22.77 ± 10.84 U/L at birth, and 19.77 ± 14.82 U/L 6 months postpartum. Upon delivery and at the 6 month visit for infants, 100% of infants had negative HBsAg and undetectable HBV-DNA levels; thus, the MTCT rate was 0% (Table 5). The immune response detected at 7–12 months postpartum was 93.4% (Table 6).

**Table 5:** Maternal treatment effects with tenofovir alafenamide fumarate.

**Variables, mean (±SD) or specified**	**Baseline = 98**	**Follow-up assessments-delivery, *N* = 98**	***P*1-values**	**Follow-up assessments-post-partum 6 months, *N* = 98**	***P*2-values**
HBV DNA changes (Log_10_ IU/mL)	7.07 ± 0.91	4.09 ± 1.12	<0.001	2.57 ± 1.87	<0.001
HBeAg positivity, *n* (%)	100%	100%	1	100%	1
HBsAg positivity, *n* (%)	100%	100%	1	100%	1
ALT (U/L)	197.41 ± 114.71	22.77 ± 10.84	<0.001	19.77 ± 14.82	<0.001

*SD, Standard deviation; HBeAg, Hepatitis B e antigen; HBsAg, Hepatitis B surface antigen*.

**Table 6:** Virological features of infants from mothers with active CHB.

**Virological features**	**At birth**	**At 7–12 months**
HBsAg positive	1/98	0/98
HBeAg positive	4/98	0/98
HBV DNA detectable	0/98	0/98
Immune response^†^	–	92/98

† *Defined as Hepatitis B surface antibody (HBsAb) ≥10 mIU/ml detected at 7–12 months of age*.

Among the 98 infants in the study, 98 (100%) received the first HBV vaccine (10 ug) and hepatitis B immunoglobulin (HBIG) (100 IU) within 12 h of birth. For the second dose of HBV vaccine, 78 (79.59%) infants were injected at 1 month, 11 (11.22%) infants received injections 4–16 days after the primary 1 month schedule due to personal reasons, and 9 (9.18%) were delayed for 2–4 weeks because of health problems such as neonatal jaundice, respiratory infections, or diarrhoea. For the third dose of HBV vaccine, 69 (70.40%) infants were injected at 6 months, and 29 (29.59%) were delayed for 1–8 weeks due to personal reasons, health problems, or the influence of COVID-19.

## Discussion

In this prospective study, we aimed to investigate the efficacy and safety of TAF treatment for HBV in infants and mothers during early and middle pregnancy. To the best of our knowledge, this is the first study involving a significant number of mothers with CHB exposed to TAF in the first and second trimesters. We observed that TAF exposure during pregnancy appears to be safe for both mothers and infants during the 6–12 months follow-up period. No congenital defects or abnormal physical growth was found among infants after exposure. MTCT of HBV was completely prevented with the use of maternal TAF therapy during pregnancy in combination with standard HBV immunoprophylaxis in infants. Additionally, TAF was well-tolerated throughout pregnancy, and no major safety concerns for either mothers or infants have been reported. In summary, our study provides important evidence to support the use of TAF as an alternative to TDF in the first and second trimesters.

In China, TAF has been licenced for the treatment of CHB since December 2018. Currently, its use is increasing in the real world and it is considered an updated version or successor to TDF (22, 23) because of its non-inferiority, significantly high rate of normalisation of ALT levels, lack of resistance, and significantly better bone and renal safety (24). Considering these benefits, the use of TAF has been optimised, especially in specific patient groups at risk of renal or bone disease suitable for TAF therapy. However, there is scarce real-world data on TAF treatment for HBV in pregnant women, especially in the first and second trimesters. Currently, TAF is not recommended during pregnancy in patients with HIV and HBV infections because of limited pharmacokinetic and safety data (5, 8, 24). However, the drug instructions for TAF in China indicate its usage during pregnancy, if necessary, which enabled our investigation in a real-world setting (14).

The efficacy of TAF treatment in mothers and infants in our study was similar to that of TDF in pivotal trials and in other studies of TAF in middle-to-late pregnancy (11, 12, 15). The primary reason for initiating antiviral treatment of HBV in pregnant women is to prevent MTCT (25). Major clinical guidelines recommend the third trimester as the start date due to inadequate evidence of safety in human data evaluated in earlier pregnancy (26). There are only a few studies assessing telbivudine and lamivudine use starting in the early pregnancy, but none assessing TAF (27–29). A high viral load is independently associated with a high risk of HBV MTCT (30). In our study, TAF therapy until delivery suppressed the maternal mean (±SD) level of HBV DNA to 4.09 ± 1.12 log_10_ IU/mL with 100% (98/98) of patients achieving the target level of <200,000 IU/mL at delivery, the same rate compared with the real-world study of TDF by Wang et al. (31). These differences could be due to the different durations of TAF treatment throughout pregnancy and maternal HBV DNA levels prior to initiating TAF treatment in the different studies. No changes in the HBsAg and HBeAg status of mothers were observed during the study period, as expected, due to the short TAF treatment duration and the relatively low efficacy of oral antiviral agents for HBV seroconversion (6). Still, prevention of MTCT is the most effective way to reduce the global burden of HBV-related liver diseases (15). Our study provides evidence on the role of TAF therapy in HBV prevention and new knowledge to enhance perinatal care in mothers with CHB.

The safety of antiviral therapy in mothers and infants is a priority. However, no data exist to support the safety profile of TAF initiated in early and middle pregnancy. In our study, the other purpose of TAF treatment initiation during pregnancy was to control maternal liver disease. No viral breakthrough or ALT flares occurred during pregnancy and follow-up periods. Twelve mothers enrolled in our study initiated TAF treatment before they were aware of the pregnancy, and they did not want to change to other antiviral drugs. In real-world clinical practise, an increasing number of Chinese women with active HBV infection require TAF for treatment. As in China, an increasing number of women of childbearing age are facing the dilemma of antiviral drug selection during pregnancy and breast-feeding periods. In our study, the maternal adverse effects and important laboratory results were critically monitored and were similar to those of other studies involving TDF and TAF in middle to late pregnancy (14, 15, 19, 31, 32). Moreover, no significant safety concerns and growth development were identified among the 98 infants enrolled in our study during the 6 month follow-up period. These results suggest that early initiation of TAF during pregnancy in mothers with active CHB is safe.

Our study had several limitations. First, this was a single-centre study. Second, TDF or other antiviral drugs were not included as a control group in our study. However, there has been plenty of studies reporting on TDF in pregnancy. Third, there is a potential selection bias for mothers due to age and drug cost. Fourth, the follow-up duration in the current study was within 6 months. Fifth, it is difficult to define that pregnant females suffered from active hepatitis based on one or two ALT report as it also happens in seroconversion (HBV related flares which do not require treatment), and some of mothers may have pregnancy related causes of high ALT. Thus, the long-term effects of foetal exposure to TAF have not yet been investigated. Therefore, future multicentre studies with larger sample sizes, control groups, and longer follow-up periods are needed to further prove the safety and efficacy of TAF, either initiated in early pregnancy for mothers with active CHB or used during the entire pregnancy for mothers with accidental pregnancy while on TAF treatment.

In conclusion, initiating TAF in early and middle pregnancy seems to be safe for both mothers and infants, and TAF is effective for controlling the maternal disease as well as preventing MTCT.

## Data Availability Statement

The original contributions presented in the study are included in the article/supplementary material, further inquiries can be directed to the corresponding author/s.

## Ethics Statement

The studies involving human participants were reviewed and approved by Institutional Ethics Review Committee of Xiangya Hospital. The patients/participants provided their written informed consent to participate in this study.

## Author Contributions

YH designed the study. JZ collected and analysed the data. RC wrote the manuscript. LL, HH, MZ, and XF collected the data. All authors have read and approved the final manuscript.

## Funding

The collection, analysis, and interpretation of data, manuscript writing, and the Rapid Service Fee for this paper was funded in full by grants from the National Natural Sciences Foundation of China (Grant Nos. 81970550 and 82070613), the National Natural Sciences Foundation of Hunan Province (Grant No. 2019JJ30041), the Innovation-Driven Project of Central South University (Grant No. 2020CX044), and the National Science and Technology Major Project of China (Grant Nos. 2018ZX10723203 and 2018ZX10302206).

## Conflict of Interest

The authors declare that the research was conducted in the absence of any commercial or financial relationships that could be construed as a potential conflict of interest.

## Publisher's Note

All claims expressed in this article are solely those of the authors and do not necessarily represent those of their affiliated organizations, or those of the publisher, the editors and the reviewers. Any product that may be evaluated in this article, or claim that may be made by its manufacturer, is not guaranteed or endorsed by the publisher.
